# Protective effects of *Sonchus asper* against KBrO_3_ induced lipid peroxidation in rats

**DOI:** 10.1186/1476-511X-11-164

**Published:** 2012-11-27

**Authors:** Rahmat Ali Khan, Muhammad Rashid Khan, Sumaira Sahreen

**Affiliations:** 1 Department of Biotechnology, Faculty of Sciences, University of Science and Technology Bannu, Bannu, Pakistan; 2 Department of Biochemistry, Faculty of Biological Sciences, Quaid-i-Azam University, Islamabad, Pakistan; 3 Botanical Sciences Division, Pakistan Museum of Natural History, Islamabad, Pakistan

**Keywords:** Potassium bromate, Sonchus asper, Antioxidant, FSH, DNA fragmentation, TSH

## Abstract

**Background:**

*Sonchus asper* is traditionally used in Pakistan for the treatment of reproductive dysfunction and oxidative stress. The present investigation was aimed to evaluate chloroform extract of *Sonchus asper* (SACE) against potassium bromate-induced reproductive stress in male rats.

**Methods:**

20 mg/kg body weight (b.w.) potassium bromate (KBrO_3_) was induced in 36 rats for four weeks and checked the protective efficacy of SACE at various hormonal imbalances, alteration of antioxidant enzymes, and DNA fragmentation levels. High performance chromatography (HPLC) was used for determination of bioactive constituents responsible.

**Results:**

The level of hormonal secretion was significantly altered by potassium bromate. DNA fragmentation%, activity of antioxidant enzymes; catalase (CAT), peroxidase (POD), superoxide dismutase (SOD) and phase II metabolizing enzymes viz; glutathione reductase (GSR), glutathione peroxidase (GSHpx), glutathione-S-tansase (GST) and reduced glutathione (GSH) was decreased while hydrogen per oxide contents and thiobarbituric acid reactive substances (TBARS) were increased with KBrO_3_ treatment. Treatment with SACE effectively ameliorated the alterations in the biochemical markers; hormonal and molecular levels while HPLC characterization revealed the presence of catechin, kaempferol, rutin and quercetin.

**Conclusion:**

Protective effects of *Sonchus asper* vs. KBrO_3_ induced lipid peroxidation might be due to bioactive compound present in SACE.

## Background

Potassium bromate (KBrO_3_) is found in drinking water, food additive [1] and hair solution [2], cause sensorineural hearing loss and adverse effects on the vestibuloocular reflex system [3,4]. KBrO_3_ induces chromosomal aberration and promoting tumorigenesis [5] due to elevation of ONOO- level and 8-hydroxydeoxyguanosine levels in DNA induced by oxidative stress [6]. Sever damage to sperm membranes, proteins and DNA is associated with alterations in signal transduction mechanisms that affect fertility [7,8]. Sertoli cells support the moment of sperm from testis into epididymis through efferent ducts [9]. Free radicals cause alterations in the spermatogenic cycle, hypogonadism, reduction of sperms, alterations of reproductive hormones and degeneration in seminiferous tubules [10]. 4–5 g *Sonchus asper* (L.) Hill powder is traditionally used in the treatment of human reproductive disorder [11], cardiac dysfunction [12] and cancer [13]. Phytochemical study revealed that *Sonchus asper* contains flavonoids glycosides, ascorbic acid and carotenoids, possess antioxidant, anticancer; anti-inflammatory properties. The present investigation was aim to conform the traditional use of *Sonchus asper* versus KBrO_3_ induced hormonal dysfunction, injuries and lipids peroxidation in rat testicular tissues.

## Materials and methods

### Plant collection

*Sonchus asper* at maturity was collected from District Bannu (Pakistan) during the September 2011, identified and a voucher specimen R-47 was submitted at herbarium of Pakistan, Quaid-i-Azam University Islamabad, Pakistan. All parts of the plant (leaves, stem, flowers, seeds and roots) were shade dried for two weeks, chopped, and ground mechanically.

### Preparation of plant extract

2 kg powder of *Sonchus asper* was extracted in 5 L chloroform to get chloroform extract (SACE). The extract was cooled at room temperature, filtered and evaporated under reduced pressure through rotary evaporator. The extract was stored at 4°C for *in vivo* investigations.

### High performance liquid chromatography of plant extract

250 mg plant powder was extracted with 10 ml of 25% hydrochloric acid and 25 ml chloroform for 1 h. The obtained extract was filtered and diluted to 100 ml. 10 μl samples were injected into the HPLC Agilent HPLC system. Separation was carried out through column C18, UV–VIS Spectra-Focus detector, injector-auto sampler. Trifluoroacetic acid and acetonitrile was used as solvent with flow rate was 1 ml/min. Different standards compounds (catechin, kaempferol, rutin and quercetin) were run for comparative detection. The calibration curves were defined for each compound in the range of sample quantity 0.02-0.5 μg. All samples were assayed in triplicate.

### Animals

36 male albino rats (180–190 g) were procured from National Institute of Health Islamabad and were kept in ordinary cages at room temperature of 25 ± 3°C with a 12 h dark/light cycle. Standard laboratory feed and water was free accessed. The study protocol was approved by Ethical committee of Quaid-i-Azam University Islamabad. All these rats were used for *in vivo* screening of chloroform fraction of *Sonchus asper* against KBrO_3_-induced testicular toxicity and hormonal dysfunction in rats.

### Experimental design

To study the antioxidant effects of *Sonchus asper*, male albino rats were equally divided into 6 groups (6 rats). SACE was dissolved in DMSO while 20 mg of KBrO_3_ in water. SACE was administered after 48 h of KBrO_3_ treatment for 30 days.

Group I Control; only allowed to food and water

Group II KBrO_3_ (20 mg/kg b.w.)

Group III KBrO_3_ (20 mg/kg b.w.) + SACE (100 mg/kg b.w. orally)

Group IV KBrO_3_ (20 mg/kg b.w.) + SACE (200 mg/kg b.w. orally)

Group V KBrO_3_ (20 mg/kg b.w.) + Silymarin (50 mg/kg b.w. orally)

Group VI SACE (200 mg/kg b.w. orally)

After 24 h of the last treatment, all the animals were weighted, sacrificed; collected their blood and urine while their testis were removed, weighted, perfuse in ice-cold saline solution and treated with liquid nitrogen for further enzymatic and DNA damage analysis.

### Serum analysis of hormone

Serum level of testosterone, luteinizing hormone (LH), follicle stimulating hormone (FSH), estradiol and prolactin was estimated using RIA gamma counter through kits.

### Assessment of antioxidant enzymes

70 mg of tissue was homogenized in 10 volume of 100 mmol KH_2_PO_4_ buffer containing 1 mmol EDTA (pH 7.4) and centrifuged at 12,000 × g for 30 min at 4°C. The supernatant was collected and used for enzymatic studies. Protein concentration of tissue supernatant was determined by the method of using crystalline BSA as standard. Various antioxidant enzymes including CAT [14], POD [15], SOD [16], TBARS [17], GST [18], GSR [19], GSHpx [20], GSH [21] and DNA fragmentation [22] were carried out.

### Histopathological overview of testis

After weighting the portion specifies for histology; testis was fixed for 3–4 h in fixative sera followed by dehydration with ascending grades of alcohol (80%, 90%, and 100%) and transferred in cedar wood oil, when testis becomes clear then embedded in paraplast and prepared blocks for further microtomy. 3–4 μm thin slides were prepared with microtome; wax was removed, stained with hemotoxilin-eosin and photographed under light microscope at 40x.

### Statistical analysis

To determine the treatment effects one way analysis of variance was carried by computer software SPSS 13.0. Level of significance among the various treatments was determined by LSD at 0.05% level of probability.

## Results

### HPLC characterization of polyphenolic constituents

**Figure 1:**
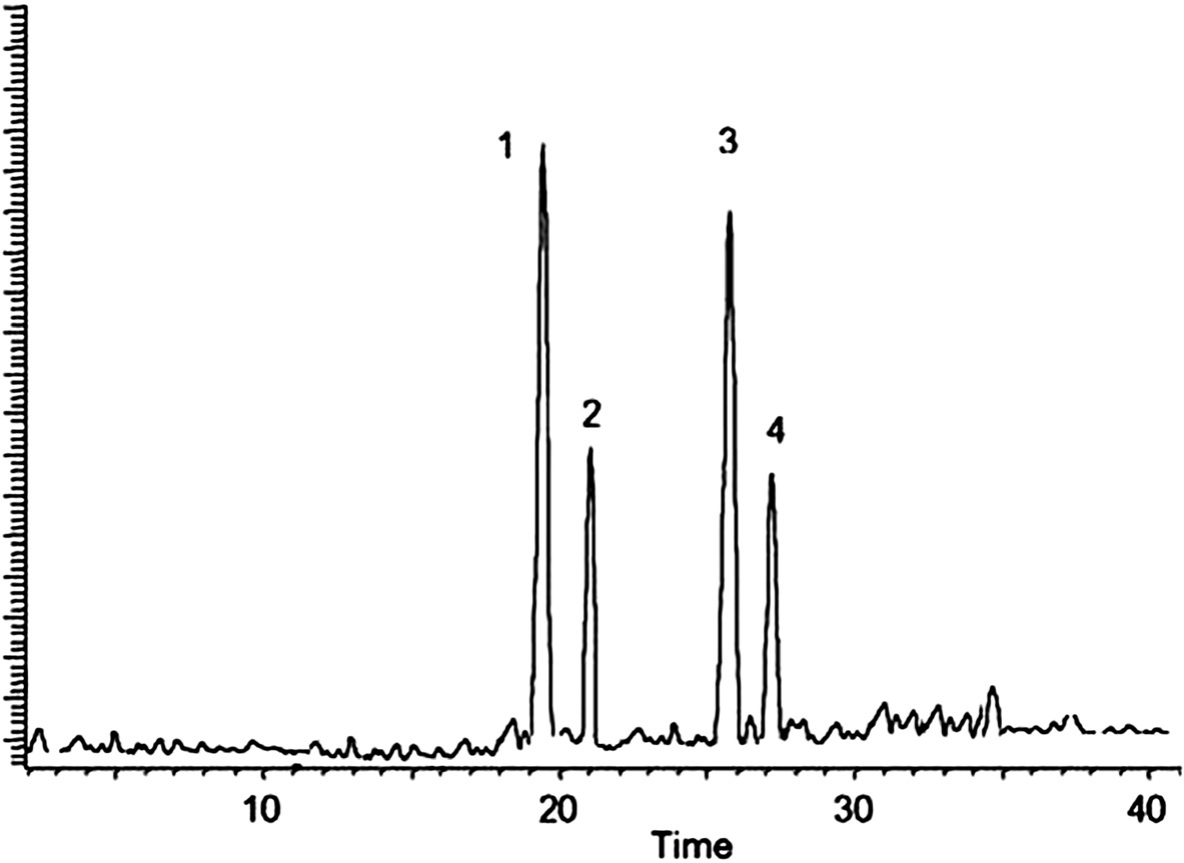
**HPLC fingerprints obtained by chloroform extract of *Sonchus asper *eluted with mixtures of trifluoroacetic acid and acetonitrile indicated the presence of 4 compounds kaempferol, rutin, catechin and quercetin.**

The investigated compounds in the SACE were quantified by integration of the peak-areas at 220 nm using an external calibration method. Least-squares linear regression was used to determine the calibration parameters for each of standards. The main polyphenolic flavonoids compounds in the SACE include catechin, kaempferol, rutin and quercetin (Figure 1).

### Effect of SACE on pituitary—gonadal axis

The mean values of the serum hormones; etradiol, prolactin, testosterone, luteinizing hormone and follicle stimulating hormone were shown in Table 1. Treatment of rats with KBrO_3_ for 4 weeks, significantly decreased *(P < 0.01)* the hormonal level of testosterone, luteinizing hormone and follicle stimulating hormone while increased markedly (*P < 0.01*) the secretion of estradiol, prolactin as against the control group. These alterations of hormones are significantly reversed (*P < 0.01)* by administrations of 100 mg/kg and 200 mg/kg b.w., SACE as well as 50 mg/kg b.w. Silymarin in KBrO_3_ treated rats however non significant changes were observed in non treated SACE alone rats.

**Table 1:** Effect of SACE on FSH, LH, testosterone, prolactin and estradiol in rat

**Treatment**	**FSH**	**LH**	**Testosterone**	**Prolactin**	**Estradiol**
	**(mg/dl)**	**(mg/dl)**	**(mg/dl)**	**(mg/dl)**	**(mg/dl)**
Control	21.5 ± 0.3++	25 ± 1.72++	47 ± 3.04++	24.5 ± 2.5++	41.0 ± 2.1++
20 mg/kg KBrO_3_	11.2 ± 0.2**	10.5 ± 0.71**	23 ± 2.7**	48.5 ± 2.7**	70.5 ± 3.0**
50 mg/kg Silymarin + KBrO_3_	19.1 ± 0.74++	20.7 ± 2. 4++	30.3 ± 3. 9**++	23.5 ± 3.09**++	51.3 ± 2.4++
100 mg/kg SACE + KBrO_3_	12.8 ± 0.12**	18.8 ± 2.04**	34.7 ± 2.53++	30.7 ± 1.7**++	53 ± 2.6**++
200 mg/kg SACE + KBrO_3_	19.2 ± 0.80++	21 ± 1.85++	36 ± 2.53++	28.5 ± 1.51*++	48.3 ± 3.01++
200 mg/kg SACE alone	24.0 ± 1.9++	24.0 ± 1.62++	45.7 ± 3.52++	23.7 ± 1.0++	40.0 ± 2.1++

Mean ± SE (n = 6 number), *, ** indicate significance from the control group at *P <* 0.01 probability level, ++ indicate significance from the KBrO_3_ group at *P <* 0.01 probability level.

### *Sonchus asper* and antioxidant profile

The results regarding the protective effects of SACE against the toxic affect of KBrO_3_ in rat on activities of antioxidant enzymes such as CAT, POD, SOD and amount of tissue protein were shown in Table 2. Activities of antioxidant enzymes such as CAT, POD, SOD and amount of tissue protein were significantly *(P < 0.01)* reduced by treatment of KBrO_3_ as compared to control group. This reduction was improved significantly *(P < 0.01)* by post-administration of 100 mg/kg and 200 mg/kg b.w. SACE and 50 mg/kg b.w. silymarin to control rat. However, non significant changes *(P > 0.05)* were found by administration of SACE alone against the control group.

**Table 2:** Effect of SACE on tissue protein, activity of CAT, POD and SOD

**Treatment**	**Protein**	**CAT**	**POD**	**SOD**
	**(μg/mg tissue)**	**(U/min)**	**(U/min)**	**(U/mg protein)**
Control	2.9 ± 0.09++	8. 9 ± 1. 8++	11.9 ± 2.17++	22.29 ± 2.07++
20 mg/kg KBrO_3_	2.07.087**	4.7 ± 0.54**	5.91 ± 0.983**	13.7 ± 1.73**
50 mg/kg Silymarin + KBrO_3_	2.82 ± 0.134++	6.804 ± 0.976++	9.45 ± 1.64*++	19.82 ± 2.31++
100 mg/kg SACE + KBrO_3_	2.97 ± 0.19**++	5.117 ± 0.575**+	7.63 ± 1.10**++	17.15 ± 1.62**++
200 mg/kg SACE + KBrO_3_	3.08 ± 0.057++	6.131 ± 0.779++	9.10 ± 1.39++	18.06 ± 2.00++
200 mg/kg SACE alone	3.52 ± 0.173++	7.38 ± 1.25++	11.50 ± 2.14++	21.52 ± 3.02++

Mean ± SE (n = 6 number), ** indicate significance from the control group at *P <* 0.01 probability level, ++ indicate significance from the KBrO_3_ group at *P <* 0.01 probability level.

### Effect of SACE on GSHpx, GST, GSR, GSH, TBARS

Effect of KBrO_3_ and the protective effects of SACE on tissue phase II metabolizing enzymes viz; GSHpx, GST, GSR, GSH and TBARS were shown in Table 3. KBrO_3_ treatment to rats significantly *(P < 0.01)* decreased the activities of GSHpx, GST, GSR and GSH while significantly *(P < 0.01)* increased the contents of TBARS in tissue homogenate as compared to control group. 100 mg/kg, 200 mg/kg b.w., SACE and 50 mg/kg b.w. silymarin showed significant protection *(P < 0.01)* and recovered the activity of enzymes near to control rat; increased the activities of GST, GSR and GSH while decreased the contents of TBARS in a dose dependent manner. SACE when administered alone did not show significant variations.

**Table 3:** Effect of SACE on testis GST, GSH-Px, GSR, GSH and TBARS in rat

**Treatment**	**GSH-Px (nM/mg protein)**	**GSR (nM/min/ mg protein)**	**GST (nM/min/ mg protein)**	**GSH (μM/g tissue)**	**TBARS (nM/min/ mgprotein)**
Control	39.44 ± 3.86++	63.44 ± 3.86++	26.44 ± 3.86++	0.875 ± 0.0894++	19.78 ± 1.18++
20 mg/kg KBrO_3_	25.25 ± 1.87**	44.25 ± 1.87**	17.25 ± 1.87**	0.570 ± 0.0443**	27.17 ± 1.92**
50 mg/kg Silymarin + KBrO_3_	34 ± 1.8++	58.2 ± 1.4++	26 ± 3.8++	0.726 ± 0.014++	20.0 ± 1.2++
100 mg/kg SACE + KBrO_3_	33.67 ± 2.14**++	53.67 ± 2.14**++	22.67 ± 2.14**++	0.757 ± 0.0523++	20.30 ± 1.27++
200 mg/kg SACE + KBrO_3_	37.83 ± 2.61++	56.03 ± 2.61++	23.03 ± 2.61++	0.797 ± 0.0611++	19.95 ± 1.20++
200 mg/kg SACE alone	41.53 ± 3.91++	64.53 ± 3.91++	27.53 ± 3.91++	0.766 ± 0.018++	19.1 ± 1.3++

Mean ± SE (n = 6 number), ** indicate significance from the control group at *P <* 0.01 probability level, ++ indicate significance from the KBrO_3_ group at *P <* 0.01 probability level.

### % DNA fragmentation, testis weight, relative testis weight

Protective effects of SACE and Silymarin against KBrO_3_ administration in rat on DNA fragmentation, testis weight and relative testis weight were shown in Table 4. Administration of KBrO_3_ significantly increased *(P < 0.01)* DNA fragmentation, testis weight and relative testis weight when compared to control group. Post-treatment with SACE and silymarin erased the KBrO_3_ toxication and significantly *(P < 0.01)* improved DNA damages, testis weight and relative testis weight kidney weight and relative tissue weight towards the control group in a dose dependent. However, non significant *(P > 0.05)* variations were observed by *S. asper* alone as compared to control group.

**Table 4:** Effect of SACE on testis weight, relative testis weight in rat

**Treatment**	**Tissue**	**Relative testis**	**%DNA**
	**weight (g)**	**Weight (g)**	**Fragmentation**
Control	6.03 ± 0.66++	0.060 ± 0.0166++	4.3 ± 0.0946++
20 mg/kg KBrO_3_	7.76 ± 0.504**	0.077 ± 0.0024**	14.0 ± 0.08**
50 mg/kg Silymarin + KBrO_3_	7.03 ± 0.008++	0.035 ± 0.0018++	6.0 ± 0.063++
100 mg/kg SACE + KBrO_3_	6.66 ± 0.26++	0.066 ± 0.0073++	8.6 ± 0.060**++
200 mg/kg SACE + KBrO_3_	6.27 ± 0.451++	0.062 ± 0.0051++	6.8 ± 0.01**++
200 mg/kg SACE alone	5.95 ± 0.189++	0.03590.00189++	4.33 ± 0.06++

Mean ± SE (n = 6 number), ** indicate significance from the control group at *P <* 0.01 probability level, ++ indicate significance from the KBrO_3_ group at *P <* 0.01 probability level.

### Effect of SACE on histopathology of testis in rats

**Figure 2:**
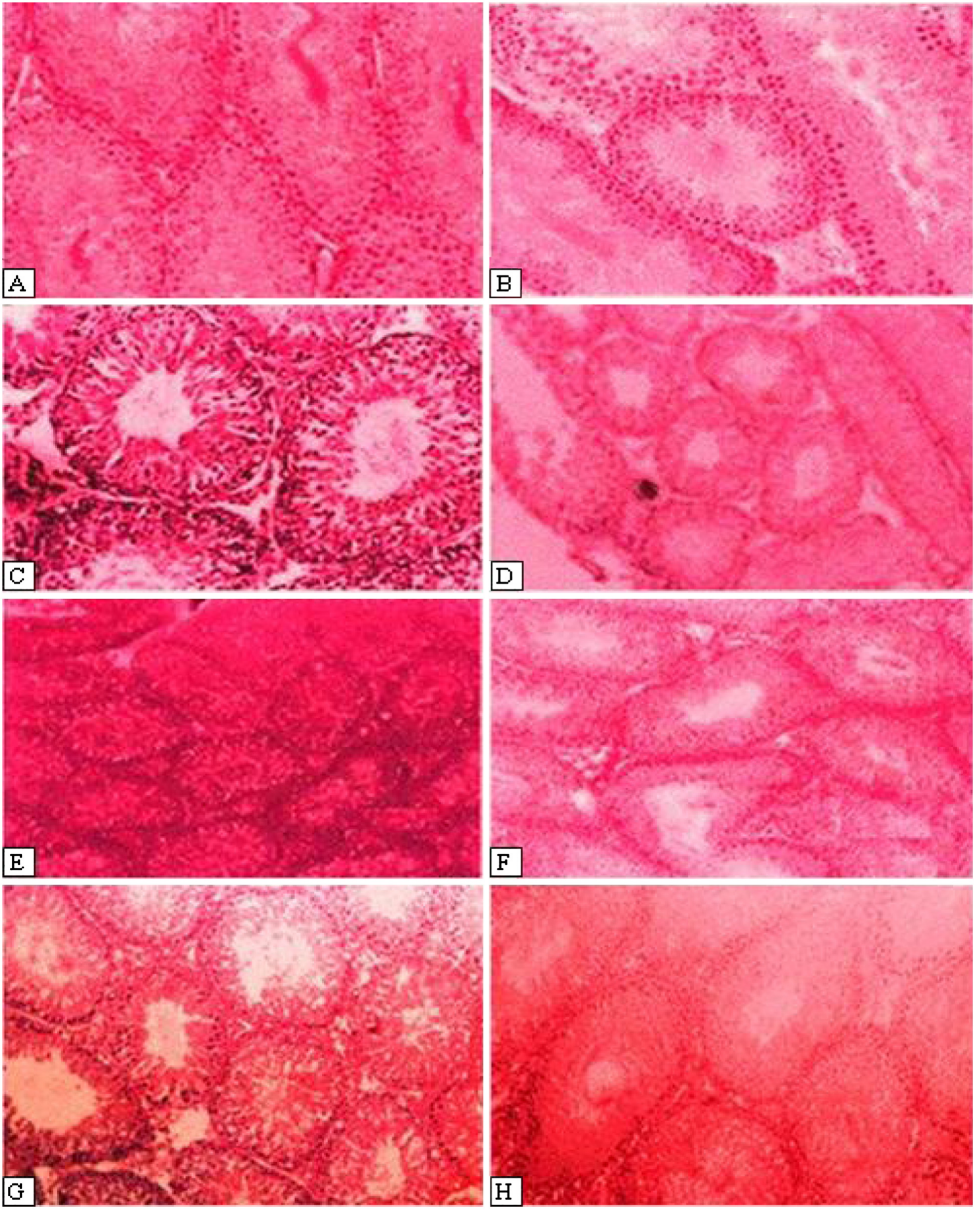
**Histopathological changes caused by KBrO**_**3 **_**and preventive effect of SACE on testis in different groups; Slides (from left) Control (A, B), KBrO**_**3 **_**(C, D), 100 mg/kg SACE + KBrO**_**3 **_**(E), 200 mg/kg SACE + KBrO**_**3 **_**(F), 200 mg/kg SACE alone (G) and 50 mg/kg silymarine + KBrO**_**3 **_**(H).**

Microscopic examinations of male reproductive system from control group revealed the normal semeniferous tubules, sperms with normal morphology and concentration (Figure 2). Sertoli cells were inconspicuous. Histological appearance of prostate glands and surrounding fibro muscular stroma was found to be normal in appearance. Administration of KBrO_3_ caused degeneration of semeniferous tubules, loss of germ cells, abnormality of germinative epithelium, interruption in meiosis, and sperm with abnormal shape and concentration were visible. Orally-administration with SACE and silymarin revealed a marked repairing of testicular abnormalities induced by KBrO_3_; sperm with normal morphology and concentration near to control group were seen in repaired semeniferous tubules (Table 5).

**Table 5:** Effect of SACE on testicular histopathology in rat

**Treatment**	**Seminiferous tubules degeneration**	**Meiosis interruption**	**Sperm concentration**	**Germ cell morphology**	**Germinative epithelium**
Control	-	-	-	**-**	-
20 mg/kg KBrO_3_	+++	++	+++	++	++
50 mg/kg Silymarin + KBrO_3_	−/+	-	-	−/+	-
100 mg/kg SACE + KBrO_3_	−/+	-	−/+	−/+	−/+
200 mg/kg SACE + KBrO_3_	-	-	-	-	−/+
200 mg/kg SACE alone	-	-	-	-	-

-, normal; −/+, mild; ++, medium; +++, severely damaged.

## Discussion

Phenolic and polyphenolic compounds are potential functional foods or neutraceuticals possess a variety of biological activities and considered to detoxify oxidative stress [23,24]. Chromatogram of the present extract showed the presence of catechin, kaempferol, rutin and quercetin. The present investigation describes molecular and biochemical processes which may be involved in the toxicity and carcinogenicity of potassium bromate [25]. In this investigation, body weight, testis weight and relative testis weight changed significantly in KBrO_3_ treated rats, in agreement with previous studies using the same dose of 80 mg KBrO_3_/kg b.w. It has been shown that increased testis weight reflects the increment of lipidperoxidation that in turn related to fatty accumulation and alteration of weight [26]. The treatment with SACE effectively abolished this change since the values were not significantly different from those of the control group, suggesting that these fractions can prevent the toxic effects of KBrO_3_ in the testis. Antioxidant enzymes play important role in detoxification of oxidative damages constitute a mutually supportive team of defense against reactive oxygen species [27]. Present study revealed that the activities of antioxidant enzymes were significantly reduced with intoxication of KBrO_3_ in rats which might be due to the presence of catechin, kaempferol, rutin and quercetin which, propagating free radicals like peroxyl radicals and converting the reactive free radicals to inactive products. Similar results were recorded by other studies of during characterization of *Digera muricata* and *Launaea procumbens* in rats [28,29]. Glutathione and other sulfhydryls play a major role in the metabolism and excretion of bromate in the rat [30]. Thiol-mediated oxidation of DNA by bromine oxides and bromine radicals has been well characterized *in vitro* and are thought to play a role in DNA damage *in vivo*[31]. While extra cellular pools of glutathione may be important in protecting target organs from bromate uptake and oxidative DNA damage, intracellular glutathione may facilitate the formation of DNA reactive metabolites [32-35]. This decrease in antioxidant status permits further oxidation of cellular DNA resulting in the formation of the mutagenic lesion 8-oxodeoxyguanosine (8-oxodG) and an increase in cellular proliferation in the target tissue. More recent evaluations of oxidative DNA damage in response to acute bromate exposures have demonstrated 8-oxodG formation in rat kidney at doses as low as 80 mg/kg [36].

The successful and complete male germ cell development is dependent on the balanced endocrine interplay of hypothalamus, pituitary and testis. Gonadotropin releasing hormone (GnRH) secreted by the hypothalamus elicits the release of gonadotropins, i.e., FSH and LH from the pituitary gland. FSH stimulates spermatogenesis while LH stimulates the production of testosterone in Leydig cells, which in turn may act on the sertoli cells and peritubular cells of the seminiferous tubules and stimulates spermatogenesis [36]. In the present study, KBrO_3_ treatment decreased the serum level of testosterone, FSH and LH. Secretion of testosterone is probably impaired due to excessive oxidative stress and the degeneration of Leydig cells [37]. Toxic effects of KBrO_3_ may result in the failure of pituitary to secrete FSH and LH and that will result in testicular dysfunction leading to infertility. In the present study, estradiol and prolactin serum level are appeared to be positively associated in the control and all the treated groups but probably the direct stimulation of the pituitary by estradiol is only one of the factors determining prolactinemia, with hypothalamic dysfunction being associated, as observed in hypogonadism. This mechanism may be considered partly responsible for the central origin of hypogonadism in our study. Spermatogenesis is a complex differentiation process. Deterioration of spermatogenesis is an integral and important part of normal spermatogenesis [8]. However, spermatogonial degeneration can result from exposure to toxic chemicals [38-40]. Result of the present study revealed that KBrO_3_ intoxication caused marked tubular degeneration, meiotic interruption, depletion of sperm concentration and degradation of germinative epithelium were confirmed. Supplementation of SACE significantly improved the testicular injuries and reversed the cellular toxicity near to control rat [41].

## Conclusion

*Sonchus asper* significantly improved the alteration of antioxidant status, DNA fragmentation and hormonal imbalance caused by KBrO_3_ in male rat which might be associated due the presence of catechin, kaempferol, quercetin and rutin. This study substantiated the scientific evidence in favors of its pharmacological use in male sexual dysfunction and hormonal imbalance in folk medicine.

## Competing interests

The authors declare that they have no competing interests.

## Authors’ contributions

RAK made significant contribution to acquisition and interpretation of data, conception and drafting of the manuscript. MRK and SS has made substantial contribution to conception and design, interpretation of data and drafting for intellectual content. All the authors read and approved the final manuscript.
